# Identification of novel metagenomic lipases through integrated structural and sequence-based analysis

**DOI:** 10.7717/peerj.20462

**Published:** 2026-02-02

**Authors:** Nongluck Jaito, Nattha Kaewsawat, Kamollak Sangawthong, Tanaporn Uengwetwanit

**Affiliations:** Center for Genetic Engineering and Biotechnology (BIOTEC), National Science and Technology Development Agency (NSTDA), Pathum Thani, Thailand

**Keywords:** Structural similarity, Metagenomics, Lipase

## Abstract

Enzymes, as key biocatalysts, are essential for advancing sustainable green technologies across diverse industrial sectors. The discovery of novel enzymes is essential for expanding their applications. In this study, we identified new lipases using an integrated screening strategy. This approach combines both structural and sequence-based methods on a large-scale metagenomic database. This strategy enabled the identification of new lipases with low sequence identity to known reference proteins. Our approach, therefore, circumvents the limitations of traditional sequence-only methods, which often fail to identify functionally similar enzymes with low sequence similarity. We first used Foldseek, a state-of-the-art structural homology search tool, to rapidly screen the database for proteins with structures similar to widely used lipases. This was followed by a rigorous sequence similarity filtering against public protein databases, yielding 711 putative novel lipases. We selected and experimentally validated three candidates, confirming their lipase activity. Further biochemical characterization revealed their notable properties including thermostability with optimal activity at 50–55 °C, and distinct alkaline activity profiles, maximal at pH of 8.0–9.0. Their unique properties, including high activity at elevated temperatures and alkaline pH, suggest potential for applications in detergent formulations, bioremediation, and industrial biocatalysis. Beyond identifying these promising enzymes, this study demonstrates the power of a combined structural and sequence-based approach for finding novel biocatalysts. This methodological innovation has broad implications for future enzyme discovery from metagenomic resources.

## Introduction

The utilization of enzymes as green catalysts has become a highly valuable technology for developing environmental sustainability and eco-friendly industrial practice (Bell et al., 2021). Enzymes can accelerate reaction rate under mild conditions with high level of substrate selectivity (O’Connell et al., 2024). Enzymes not only enhance catalytic efficiency but also reduce waste generation (Lozano & García-Verdugo, 2023). Consequently, the utilization of biocatalysts is steadily increasing as manufacturing shifts towards a circular economy and climate change mitigation. The global industrial enzymes market was valued at $7.95 billion in 2024 and is projected to reach approximately $15.50 billion by 2034 (Precedence Research, 2024). As industries continue to evolve, the search for novel enzymes remains essential.

Lipases are versatile enzymes with a wide range of industrial applications. Their broad applicability comes from their diverse properties and their ability to catalyze a variety of reactions including hydrolysis, esterification, transesterification, and alcoholysis (Vardar-Yel, Tütüncü & Sürmeli, 2024). While lipases can be sourced from animals and plants, microbial lipases are preferred for industrial applications. Microbial lipases offer a higher production yield, shorter fermentation times, and can be easily genetically modified for enhanced performance (Ali et al., 2023; Chandra et al., 2020; Xu et al., 2025). These advantages make them a more cost-effective and superior source for large-scale production. Additionally, microbial lipases have gained attention for their ability to function under extreme conditions, stability in organic solvents, and high chemo- and enantioselectivity (Vardar-Yel, Tütüncü & Sürmeli, 2024). For example, lipases in detergent formulations needs to be stable across a wide range of pH and temperatures, and compatible with detergent components such as surfactants, oxidizing agents, and metal ions (Devi et al., 2025; Safdar, Ismail & Imran, 2023). In contrast, chemical synthesis and pharmaceutical applications demand lipases with high substrate selectivity (regio-, stereo-, and enantioselectivity). This precision is necessary to synthesize specific stereoisomeric compounds (Jaito et al., 2024; Khanra, Ravichandiran & Swain, 2025; Noro, Cavaco-Paulo & Silva, 2022). Similarly, substrate-specific lipases are essential for producing desirable volatile flavored components in food industry (Lampi et al., 2020; Vélez et al., 2023). For biodiesel production, thermostable and organic solvent-tolerant lipases are preferred to efficiently catalyze the esterification reactions (Riyadi et al., 2024; Salihu & Alam, 2015). Given that each application demands specific lipase characteristics, the discovery of novel enzymes is crucial across various biotechnological fields.

Most industrial enzymes come from cultivated microorganisms. However, this source represents only a fraction of total microbial diversity. Approximately 99% of environmental microbes remain uncultivable under standard laboratory conditions (Dai et al., 2025; Jiao et al., 2021). Metagenomics offers a solution, enabling the discovery of novel enzymes directly from environmental samples without the need for cultivation (Molina-Espeja et al., 2023; Robinson, Piel & Sunagawa, 2021). Moreover, large publicly available metagenomic datasets from diverse environments could be utilized. Annotation of metagenomic data commonly relies on sequence-based methods (Ariaeenejad et al., 2024; Fulke et al., 2024; Hera et al., 2024). Sequence homology searches have been successful in discovery of several novel enzymes (Ariaeenejad et al., 2024; Dong et al., 2024; Yu et al., 2023). However, they have inherent limitations. This poses a significant challenge when attempting to identify enzymes with low sequence identity, which is often referred to as the twilight zone (Kabir et al., 2024).

Protein structure is generally more conserved than its sequence (Pereira et al., 2025; Puente-Lelievre, Malik & Douglas, 2025). Therefore, a structural similarity search can identify evolutionarily distant proteins that share the same fold. This approach is valuable for finding proteins with potentially similar functions, even when their amino acid sequences are too dissimilar to provide meaningful clues. This allows us to uncover novel enzymes that would be missed by sequence-only methods. Traditional structure comparison is computationally intensive. Despite this, recent advances in algorithms like Foldseek (Van Kempen et al., 2024) make it possible. These advances allow massive metagenomic datasets to be screened with high sensitivity and speed, unlocking the potential to discover a wider range of novel enzymes.

In this study, we explored identifying novel lipases from metagenomic data using structure- and sequence-based search methodologies. These novel lipases were defined as having no matches to existing annotated sequences. Out of 711 potential new lipases, three enzymes were selected for experimental validation and enzymatic characterization. By utilizing structural similarities instead of solely relying on sequence homology, we could effectively identify enzymes that would otherwise remain undiscovered by sequence-based methods. This would be highly valuable as biocatalysts in various industrial applications.

## Materials & Methods

### Structural and sequence similarity search

To screen lipases based on structural similarity search, we used lipase structures from Protein Data Bank (PDB) (Berman et al., 2000) as queries. These included lipases from *Aspergillus oryzae* (PDB ID: 5XK2), *Bacillus subtilis* (PDB ID: 2QXT), *Burkholderia glumae* (PDB ID: 1TAH), *Candida antarctica* (currently named *Moesziomyces antarcticus*, PDB ID: 4ZV7), *Rhizopus niveus* (PDB ID: 1LGY), and *Thermomyces lanuginosus* (PDB ID: 1DT3). Foldseek webserver was employed to search against MGnify-ESM30-v1 (Van Kempen et al., 2024). The Mgnify is non-redundant protein database predicted from metagenomic assemblies (Richardson et al., 2023). In addition to alignment results, predicted three-dimensional structures were also downloaded. With default parameters of Foldseek, structural matches can yield up to 2,000 hits. We filtered Foldseek results to include only those with query coverage of ≥50%. The results from each query for the same enzyme were combined, and redundant sequences were removed. The obtained sequences were further annotated with OmicsBox v3.3.2 (BioBam Bioinformatics, 2024; Luan et al., 2021). Sequence alignment was carried out with Diamond (Buchfink, Xie & Huson, 2015) against RefSeq release 223 database (O’Leary et al., 2016) with expectation value cutoff of 10^−15^. After initial structural similarity screening, potential novel lipases were identified based on their unique sequences (*i.e.,* sequences with no matches to existing protein sequences in the database). These sequences were then further analyzed to identify common lipase motifs including GXSXG and TWSQG. In these motifs, X represents any amino acid (Derewenda & Derewenda, 1991; Uppenberg et al., 1994). Candidate structures were visualized and inspected using BIOVIA Discovery Studio (Dassault Systemes, 2024). Structural similarity was determined by employing a pairwise structure alignment tool (Bittrich et al., 2024). PsiPred (McGuffin, Bryson & Jones, 2000) was utilized to visualize the secondary structure elements.

### Gene construction and protein expression

The candidate lipase genes were codon-optimized for prokaryotic expression. All genes were synthesized by GenScript and initially cloned into the pET23a vector at the *BamH* I and *EcoR* I sites. The vectors were transformed into *Escherichia coli* BL21DE3. However, the expressed proteins were completely insoluble, preventing further biochemical characterization. To improve solubility, the genes were subsequently subcloned into the pMAL-c5x expression vector between the *Nde* I and *Hind* III sites and re-transformed into *E. coli* BL21DE3. Fusion with maltose-binding protein (MBP) significantly enhanced the solubility of the novel lipases, enabling their biochemical characterization. The final fusion protein constructs included an N-terminal MBP tag (42.5 kDa), the target lipase protein (estimated at 27.1 kDa for MGL-1, 28.7 kDa for MGL-2, and 27.4 kDa for MGL-3), and a C-terminal His_6_ tag (0.8 kDa). The expected molecular weight of the full fusion proteins is approximately 71 kDa. A total of 10 colonies were randomly selected for colony polymerase chain reaction (PCR) using gene-specific primers. The positive colonies were subsequently sequenced to verify the presence of the correct gene, and one confirmed clone was chosen for protein expression.

A single colony confirmed to contain the target gene was cultured in 5 mL of Luria-Bertani (LB) medium supplemented with 50 µg/mL ampicillin at 37 °C and 200 rpm. After 18 h, 500 µL of the seed culture was transferred into 500 mL (four flasks) of LB medium supplemented with antibiotic. The culture was incubated at 37 °C with shaking at 200 rpm until the cell density reached an OD_600_ of 0.4–0.6. Lipase expression was induced by adding isopropyl *β*-D-1-thiogalactopyranoside (IPTG) to a final concentration of 0.1 mM. The culture was then incubated at 16 °C for 20 h with shaking conditions. Cells were harvested by centrifugation at 8,500 rpm for 5 min at 4 °C. The supernatant was discarded, and the cell pellet was resuspended in 200 mL of 50 mM Tris–HCl buffer pH 8.0. The cells were lysed by sonication, and the lysate was separated by centrifugation at 17,000 rpm for 20 min at 4 °C to remove cell debris. The crude enzymes of lipases were applied through chromatography columns for protein purification.

### Protein purification

The crude enzyme extract (supernatant) was filtered through a 0.2 µm membrane filter. It was then loaded into a HiTrap™ Q HP anion exchange chromatography column (GE Healthcare, Chicago, IL, United States), which was equilibrated with 20 mM Tris–HCl, pH 8.0 (buffer A) at a flow rate of 3 mL/min. After washing the column with five column volumes (100 ml) of buffer A, bound proteins were eluted stepwise with increasing NaCl concentrations from 100 to 1,000 mM. Since the target protein was found in the unbound fractions, these were pooled. This pooled sample was then mixed in a 1:2 (v/v) ratio with buffer B (50 mM sodium phosphate, pH 8.0, containing 1.5 M NaCl). This sample was then applied to a hydrophobic interaction chromatography on a HiPrep™ Butyl FF column (GE Healthcare, Chicago, IL, United States) equilibrated with buffer B at flow rate 3 mL/min. The target protein was unbound protein and washed out with buffer B. Bound proteins were eluted with 50 mM sodium phosphate buffer (Na_3_PO_4_). The fractions containing target protein were then pooled and dialyzed overnight against 20 mM Tris–HCl buffer (pH 8.0) at 4 °C. Next, the dialyzed sample was applied to a HiTrap™ SP HP cation exchange chromatography column (GE Healthcare, Chicago, IL, United States). The column was equilibrated with buffer A at 1 mL/min. Unbound proteins were washed out. Bound proteins were then eluted using a stepwise increase of NaCl from 100 to 1,000 mM in buffer A. Finally, the fractions containing the target protein were pooled and dialyzed against 50 mM Tris–HCl buffer (pH 8.0).

The protein concentration of the purified lipase was determined using the Bradford method (Bradford, 1976), with bovine serum albumin (BSA) from Sigma (St. Louis, MO, USA) as the standard. For the assay, 100 µL of each protein sample was mixed with 5X Bradford reagent (Bio-Rad Laboratories, Inc., Hercules, CA, USA). The absorbance was then measured at 595 nm to quantify the protein. The purity of the lipase was assessed by sodium dodecyl sulfate-polyacrylamide gel electrophoresis (SDS-PAGE), using a modified Laemmli method (Laemmli, 1970). Gels were prepared with 12% and 4% acrylamide for the separating and stacking gels, respectively. After electrophoresis, protein bands were visualized by staining the gel with a Coomassie Blue G250 Stain Solution kit (Abcam, Waltham, MA, USA). The stained gel was then imaged using a gel documentation system (Bio-Rad Laboratories, Hercules, CA, USA).

### Determination of enzyme activity

Lipase activity was determined using a colorimetric assay based on the hydrolysis of *p*-nitrophenyl esters. The reaction mixture (1 mL total volume) consisted of 50 mM Tris–HCl buffer (pH 8.0), 0.4% (v/v) Triton X-100, 1 mM *p*-nitrophenyl hexanoate (*p*NP-C6) dissolved in acetonitrile, and 100 µL enzyme. The reaction was incubated at 50 °C for 10 min. It was terminated by the addition of 300 µL of 0.1 M Na_2_CO_3_. A 100 µL aliquot from the reaction mixture was transferred to a 96-well microplate. The absorbance was measured at 405 nm. A standard curve of *p*-nitrophenol (*p*NP) was used to calculate the amount of product released. Lipase activity was defined as the amount of enzyme required to release 1 µmol of *p*NP per minute under the assay conditions. All assays were performed in triplicate.

### Determination of lipolytic activity

Tributyrate and tricaprylin were purchased from Sigma (St. Louis, MO, USA) and TCI (Chuo-ku, Tokyo, Japan), respectively. The substrate specificity of each enzyme was investigated using a pH indicator assay modified from the previous study (Camacho-Ruiz et al., 2015; Mateos-Díaz et al., 2012). The reaction mixture (1 mL) contained 50 mM Tris–HCl buffer (pH 7.0), 100 mM NaCl, 0.42 mM p-nitrophenol, 4.2 mM substrate, and 50 µg of enzyme. The mixture was incubated at 30 °C for 10 min. The absorbance at 405 nm was measured to assess the change in color intensity. This change corresponds to the enzymatic hydrolysis of the substrate and subsequent release of fatty acids. Enzyme activity was analyzed as the percentage reduction in absorbance compared to the blank (reaction mixture without enzyme).

### Determination of hydrolysis of chain-length specificity

*p*-nitrophenyl esters with varying acyl chain lengths were investigated to evaluate chain-length specificity. The esters included *p*-nitrophenyl acetate (C2), butyrate (C4), hexanoate (C6), octanoate (C8), decanoate (C10), dodecanoate (C12), and palmitate (C16). All substrates were purchased from Sigma (St. Louis, MO, USA). The reaction mixtures included 1 mM of the above substrate dissolved in acetonitrile, 100 μL of enzyme (0.03 mg MGL-1, 0.11 mg MGL-2, 0.01 mg MGL-3), 0.4% (v/v) Triton X-100, and 50 mM of buffer. Enzyme assays were performed under the optimal temperature and pH for each enzyme. Specifically, the optimal conditions were 50 °C in Tris–HCl buffer (pH 8.0) for MGL-1; 50 °C in Tris–HCl buffer (pH 7.0) for MGL-2; and 60 °C in Glycine-NaOH (pH 9.0) for MGL-3. The experimental controls without enzyme were included using the same conditions. All reactions were performed in triplicate. The maximum activity observed was set to 100%, and all results were presented as relative activity.

### Determination of optimal temperature and pH conditions for lipase activity

The optimal temperature and pH for the hydrolytic activity of the lipase were investigated. A 1 mL reaction mixture was prepared according to the enzyme activity assay protocol. *p*-nitrophenyl hexanoate (*p*NP-C6) was used as the substrate in both the pH and temperature experiments. Each reaction mixture contained 100 μL of enzyme (0.03 mg of MGL-1, 0.11 mg of MGL-2, or 0.01 mg of MGL-3).

For optimal temperature studies, the reaction mixture was incubated for 10 min at various temperatures (25, 30, 40, 50, 60, 70, 80, and 90 °C). To evaluate optimal pH effects, different buffer systems were used including sodium acetate buffer (pH 4.0–5.0), potassium phosphate buffer (pH 5.0–7.0), Tris–HCl buffer (pH 7.0–9.0), and glycine-NaOH buffer (pH 9.0–10.0). In each sample test, the reaction mixture was incubated at the enzyme’s optimal temperature for 10 min. The reaction was terminated, and the enzyme activity was determined following the enzyme activity assay protocol. The reaction mixture without enzyme was used as a control. All experiments were repeated in triplicate. The effect of temperature and pH on lipase activity was reported by relative enzyme activity. The maximum enzyme activity was determined to be 100%.

## Results

### Structure- and sequence-based screening for novel lipases

**Figure 1:**
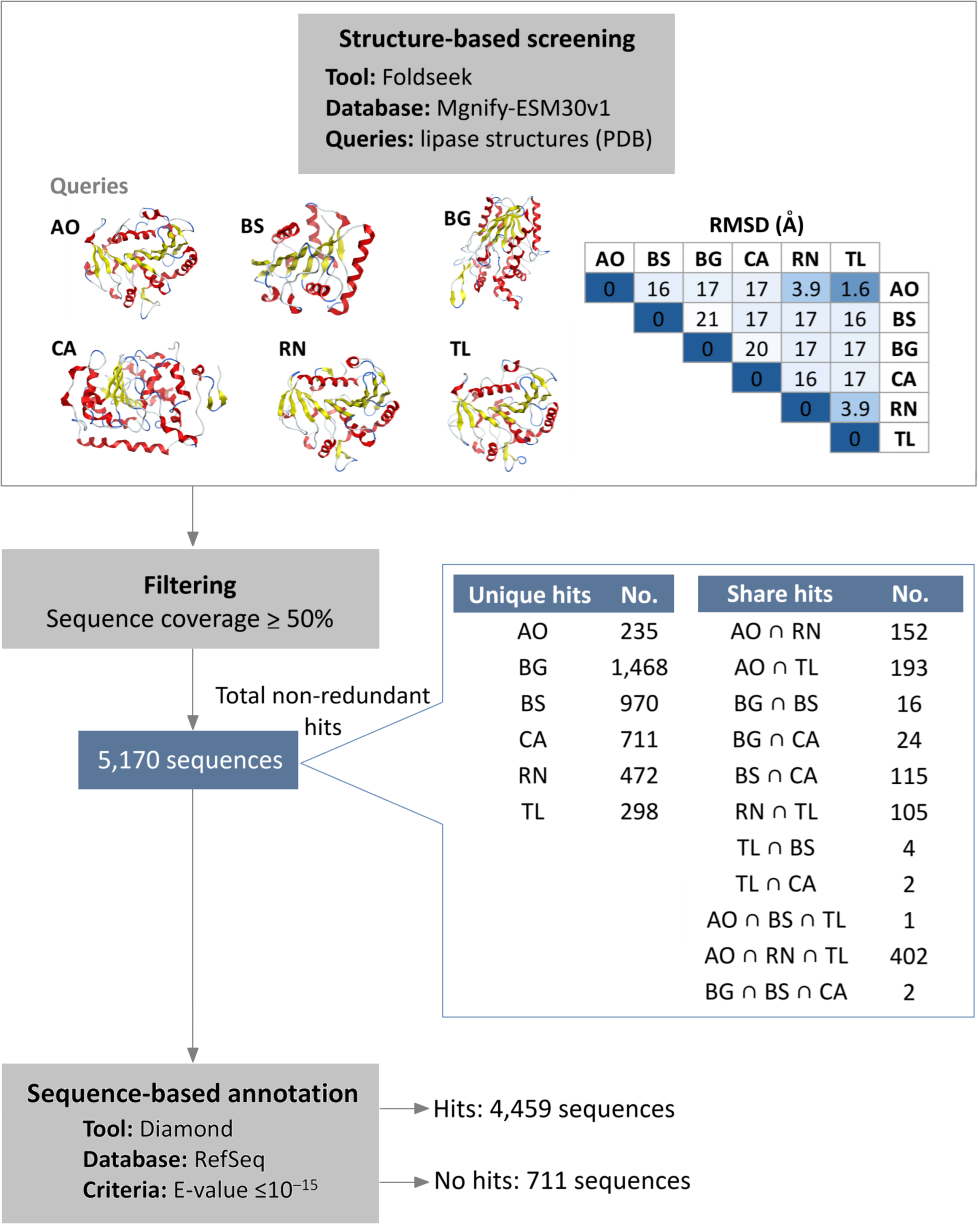
Integrated structure- and sequence-based screening workflow and results. Six lipase structures including *A. oryzae* lipase (AO), *B. subtilis* lipase (BS), *B. glumae* lipase (BG), *C. antarctica* lipase (CA), *R. niveus* lipase (RN), and *T. lanuginosus* lipase (TL) were used as queries to screen for structural similarity in this study. A pairwise comparison matrix displays the root mean square deviation (RMSD) values (in Å) in the upper triangle, showing the structural relationships among the query structures. The hits from each query were de-duplicated to yield 5,170 non-redundant sequences. These sequences were clustered to identify unique and shared proteins, with an emphasis on the 100% identical sequences found across different queries. Sequence similarity search was subsequently employed to filter out previously characterized lipases.

Initial structural screening using diverse representative lipase structures as queries in Foldseek against the Mgnify metagenomics database. This process identified 5,170 hits that met the filtering criteria of at least 50% query coverage (Fig. 1, Table S1). These hits were distributed among the lipase sources as follows: 983 from *A. oryzae* lipase (AO), 1,108 from *B. subtilis* lipase (BS), 1,510 from *B. glumae* lipase (BG), 854 from *C. antarctica* lipase (CA), 1,131 from *R. niveus* lipase (RN), and 1,005 from *T. lanuginosus* lipase (TL). Overlap was observed among hits from different queries, particularly among *A. oryzae*, *R. niveus*, and *T. lanuginosus* lipases, which exhibited small structural deviation (root mean square deviation (RMSD) values less than 4 Å). The intersection of hits from *A. oryzae*, *R. niveus*, and *T. lanuginosus* (AO ∩ RN ∩ TL) yielded 402 shared enzymes, with unique hits of 235 for *A. oryzae*, 472 for *R. niveus*, and 298 for *T. lanuginosus*.

To filter out previously characterized lipases, a subsequent sequence similarity search was conducted. The results showed that 4,459 sequences matched reference protein sequences. Among the matched sequences, 55% were identified as *α*/*β* fold hydrolases, 25% as lipases, and 9% as hypothetical proteins (Table S2). Conversely, 711 sequences did not exhibit significant similarity to known proteins, suggesting they are potential novel lipase candidates. Of these candidates, 586 are observed to have a conserved lipase motif.

**Figure 2:**
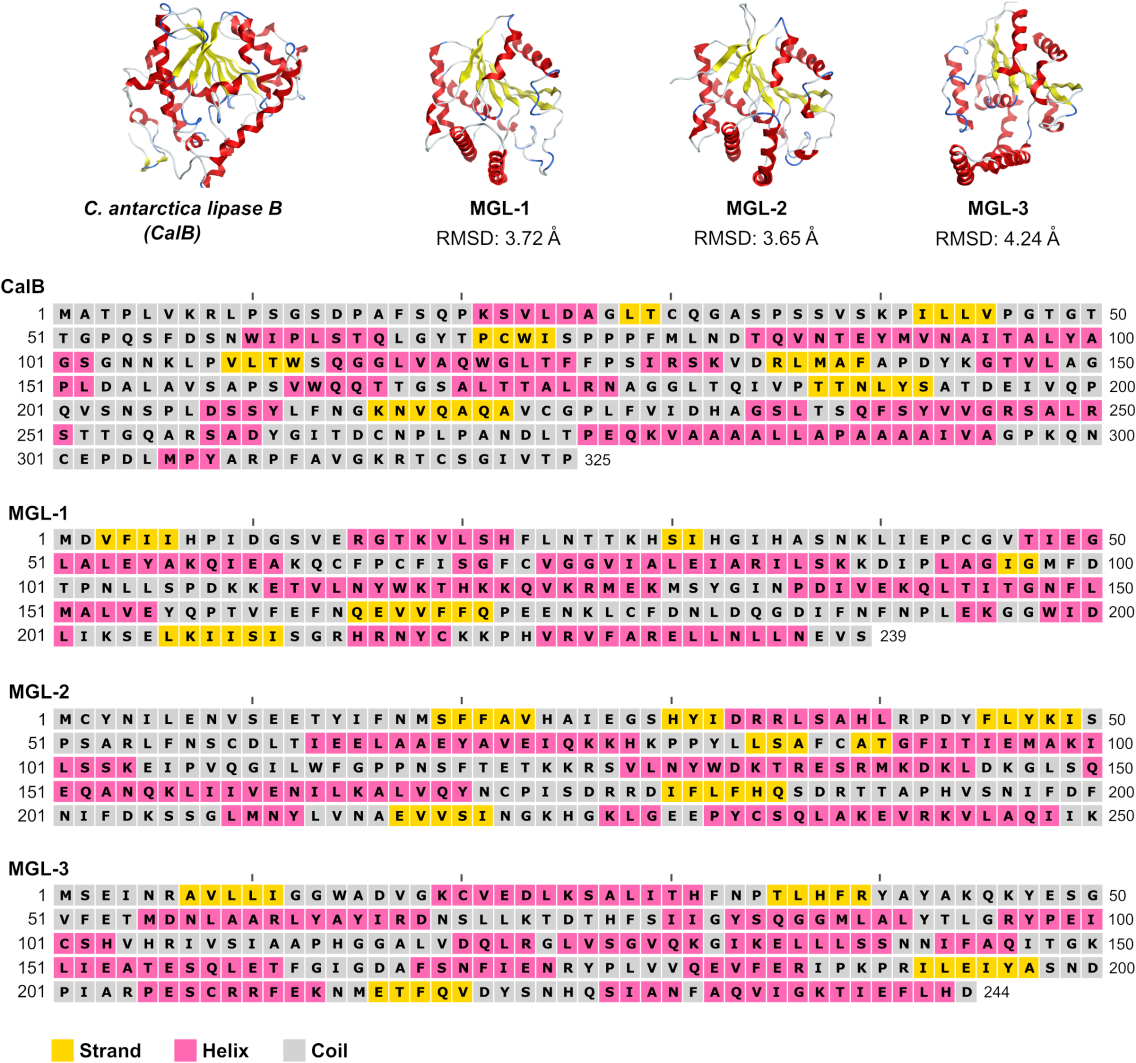
Structures and sequences of a query lipase (CalB) and candidate novel lipases.

To further validate the screening methodology and characterize novel lipases, three candidates exhibiting structural homology with *C. antarctica* lipase B (CalB) were chosen for experimental evaluation. These selected enzymes were designated MGL-1, MGL-2, and MGL-3 (Fig. 2), corresponding to MGnify IDs MGYP000002890118, MGYP000398019575, and MGYP001022591662, respectively. Their structural similarities to CalB were quantified by RMSD values of 3.72 Å for MGL-1, 3.65 Å for MGL-2, and 4.24 Å for MGL-3. Among these three, only MGL-3 was found to possess the conserved GXSXG motif, a characteristic feature of many lipases.

#### Enzymatic hydrolysis

**Figure 3:**
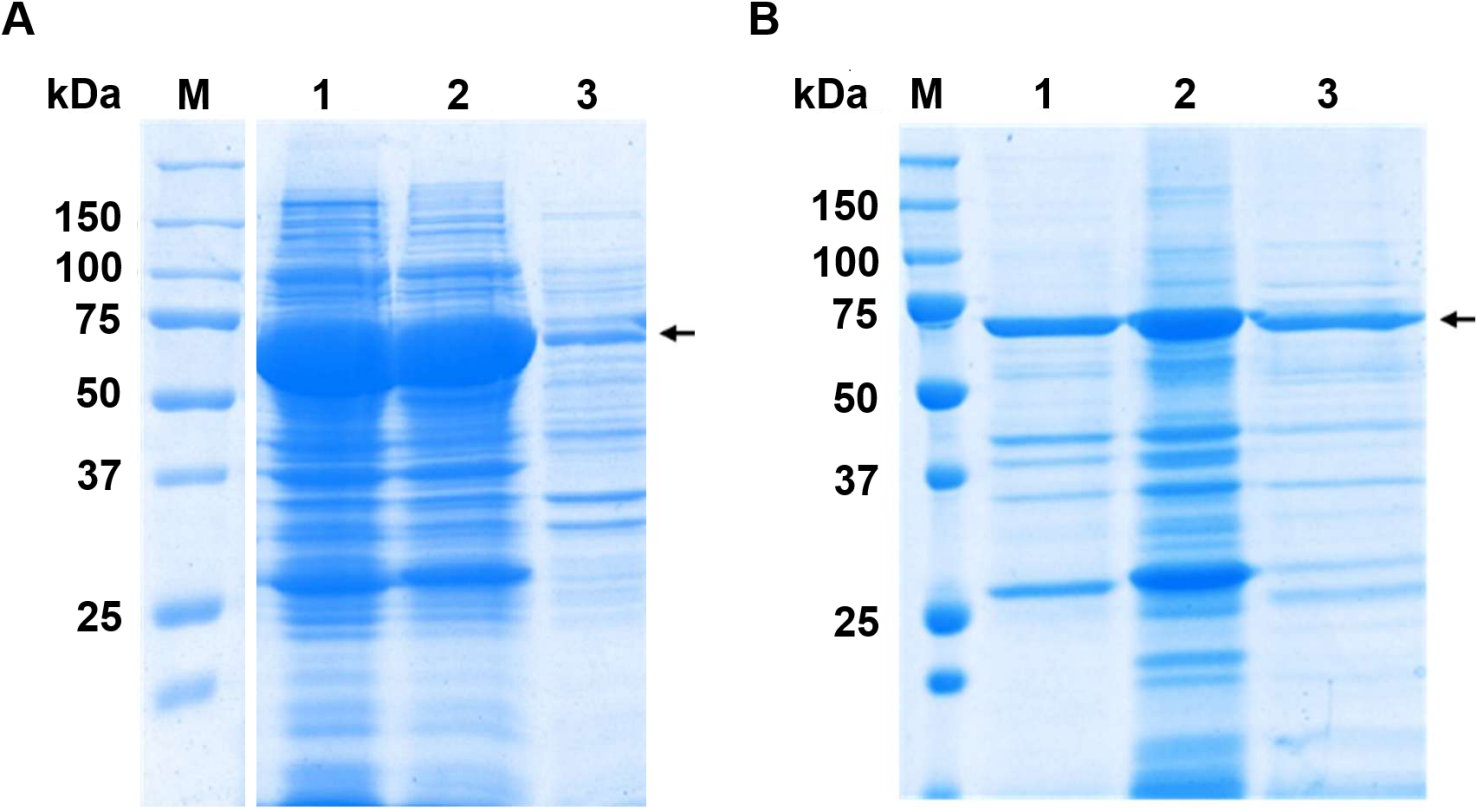
SDS-PAGE analysis before and after partial purifications. (A) Crude extract protein profiles. Raw SDS-PAGE image is provided in Fig. S1 (B) SDS-PAGE profile of partially purified lipases. Lane M is protein molecular marker (kDa). Lanes 1, 2, and 3 show the partially purified lipases MGL-1, MGL-2, and MGL-3, respectively. The target genes were engineered as fusion proteins to enhance solubility and facilitate subsequent purification. These constructs included an N-terminal maltose-binding protein (MBP) tag (42.5 kDa), the target lipase protein (estimated at 27.1 kDa for MGL-1, 28.7 kDa for MGL-2, and 27.4 kDa for MGL-3), and a C-terminal His tag (0.8 kDa). The expected molecular weight of the full fusion proteins is approximately 71 kDa. An arrow indicates the estimated molecular weight of the target lipase protein band.

The candidate enzymes, MGL-1, MGL-2, and MGL-3 were expressed as fusion protein. These fusion proteins contained N-terminal MBP tag, the respective lipases, and a C-terminal His tag, yielding an expected molecular weight of ∼71 kDa. The SDS-PAGE analysis successfully confirmed the expression and partial purification of the three candidate lipases (Fig. 3). All samples showed a major, highly enriched protein band consistent with the predicted fusion protein. Furthermore, most of the lower molecular weight contaminating proteins seen in the crude extract (Fig. 3A) were successfully removed following the partial purification step.

**Figure 4:**
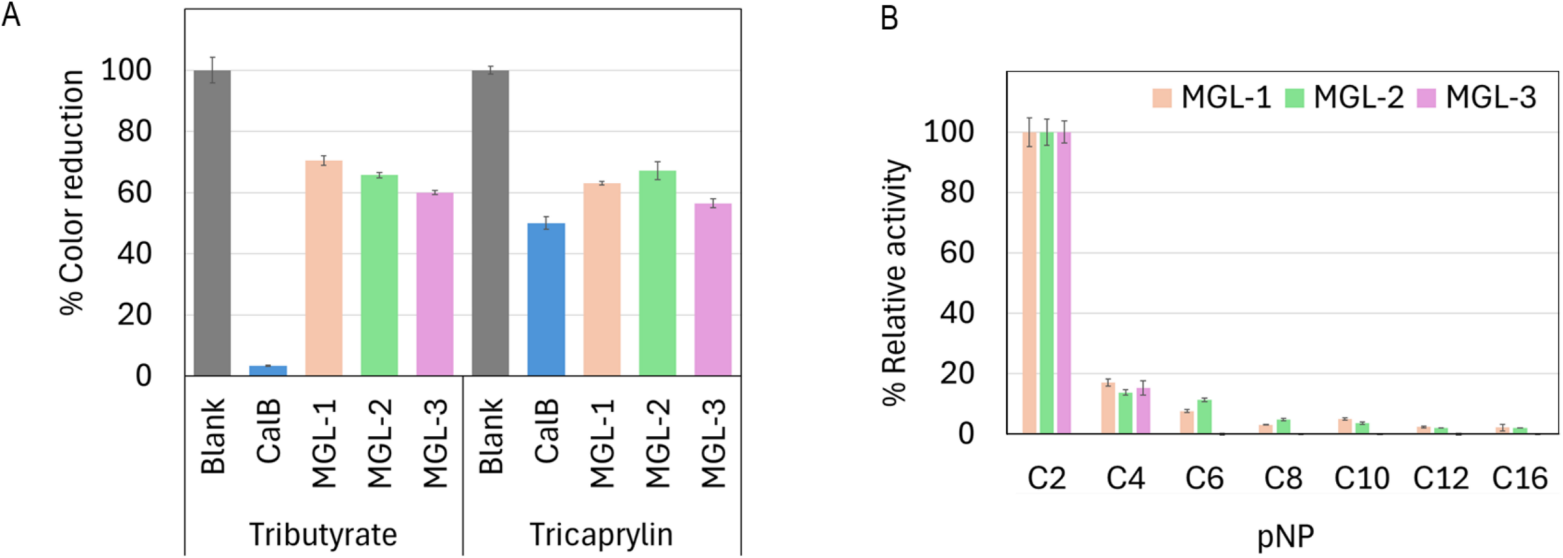
Biochemical characterization of putative novel lipases. (A) Relative lipase activity towards tributyrin and tricaprylin. (B) Substrate specificity determined using p-nitrophenyl (pNP) esters with varying acyl chain lengths. Data are presented as means ± SD.

All three enzymes showed lipase activity by hydrolyzing both short-chain tributyrin and long-chain tricaprylin (Fig. 4A). However, their overall triacylglycerol hydrolysis activity was lower than that of the reference enzyme, CalB. Further characterization of their hydrolytic activity and substrate specificity using a range of p-nitrophenyl (pNP) esters showed a strong preference for *p*NP-acetate (C2) across all candidates. This activity was notably over 5-fold higher compared to pNP-esters with longer acyl chains (Fig. 4B).

#### Optimum pH and temperature

**Figure 5:**
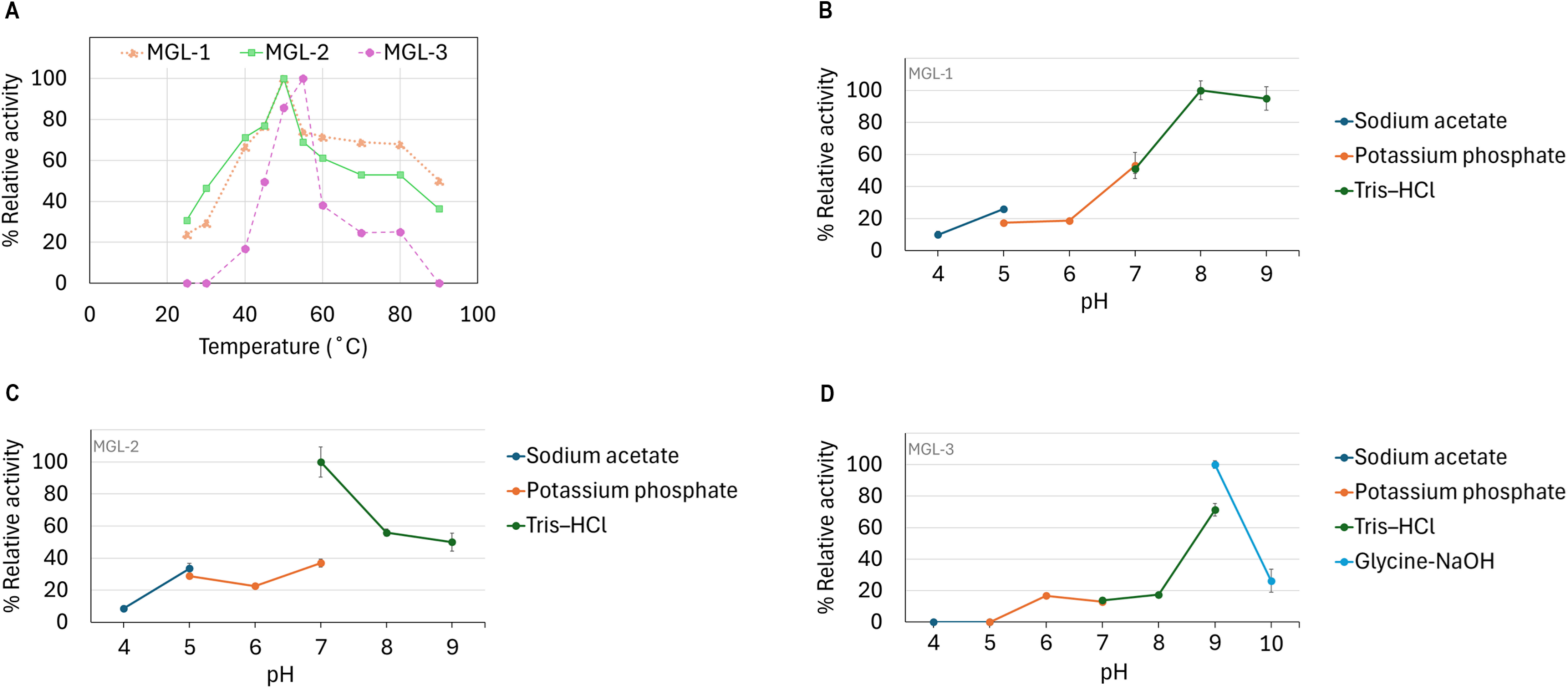
Determination of optimum temperature and pH. (A) Optimum temperature. (B) Optimum pH of MGL-1. (C) Optimum pH of MGL-2. (D) Optimum pH of MGL-3.

The candidate lipases showed distinct optimal temperature and pH profiles. MGL-1 and MGL-2 performed best at 50 °C, while MGL-3’s optimal temperature was slightly higher at 55 °C (Fig. 5A). Regarding pH, MGL-1 was most active at pH 8, MGL-2 at pH 7, and MGL-3 at pH 9 (Figs. 5B, 5C, 5D). Notably, MGL-3 was completely inactivated at pH values of 5 or lower. Differences in enzyme activity were observed for MGL-2 at pH 7 when using different buffers (potassium phosphate *versus* Tris–HCl) (Fig. 5C).

## Discussion

Lipases exhibit considerable structural diversity, which underpins their broad industrial utility (Chandra et al., 2020; Mala & Takeuchi, 2008; Puente-Lelievre, Malik & Douglas, 2025). This variability stems from their biological origin (*e.g.*, microbial, plant, or animal) and their specific physiological functions (Sharma et al., 2024). To account for this diversity, six commonly used lipases were employed as structural queries in this study. Structural search results showed that lipases with low RMSD values, indicating high structural similarity, resulted in more shared hits. For example, using lipases from *A. oryzae*, *R. niveus*, and *T. lanuginosus*, which had pairwise RMSD values <4 Å, yielded a high number of shared hits. In this study, we used Foldseek which utilizes structural alphabet describing tertiary amino acid interaction. The observed link between structural similarity and shared hit sequences suggests that Foldseek reliably and efficiently detects structural homologs.

Sequence-based searching was subsequently used to prioritize putative novel lipases. This involved filtering out sequences highly similar to previously characterized reference proteins (RefSeq). More than half of matched amino acid sequences were identified as *α*/*β* hydrolase fold proteins. The *α*/*β* hydrolase fold, characterized by a central *β*-sheet surrounded by *α*-helices, is a common protein structure found across a broad range of enzymes including lipases, esterases, and proteases (Ozhelvaci & Steczkiewicz, 2025). The presence of this specific fold suggested that these proteins could indeed be lipases. However, it is crucial to acknowledge that while protein structure is more conserved than sequence, structural similarity does not guarantee functional identity (Bzówka et al., 2022; Chen, Xie & Wu, 2017). Therefore, functional analysis and experimental characterization are essential to confirm their specific enzymatic activity.

By integrating both structural and sequence-based screening, we identified 711 putative novel lipases from the Mgnify metagenomics database. Some of these putative novel lipases showed only structural similarity to known lipases, but no sequence similarity. For these, the presence of the highly conserved lipase motif was used to assess the likelihood of the method correctly identifying true lipases. This was necessary because actual experimental validation was not feasible for all candidates. Notably, 586 out of these 711 putative novel lipases (accounting for 82.5%) possessed lipase motifs. This suggests that the integrated method is effective in screening novel enzymes.

Among the putative novel lipases, we prioritized the candidates that exhibited structural homology to CalB for experimental testing. CalB is a widely used in various industry applications due to its specificity and catalytic efficiency (Wang et al., 2023). We selected three candidates (MGL-1, MGL-2, and MGL-3). The presence of a specific lipase motif was not a criterion for candidate selection because lipases can possess diverse motifs (Cao et al., 2015; Gutiérrez-Domínguez et al., 2022).

The candidate lipases were expressed with a C-terminal His tag and an N-terminal MBP tag to enhance solubility and facilitate purification. The distinct presence and enrichment of the target fusion protein band across all samples confirmed that the partial purification step was effective. However, the degree of purification varied among the candidates. MGL-1 exhibited a higher degree of purity, evidenced by a dominant target fusion protein band and only minimal contaminating proteins. MGL-2 and MGL-3, however, were less efficiently purified. Although the target was clearly enriched, a noticeable number of contaminating proteins persisted. This difference in purification efficiency may be due to differences in stability, aggregation, or binding affinities. Nevertheless, the successful initial expression and purification enabled the subsequent biochemical characterization of all three novel lipases.

The enzymatic assays confirmed that all three candidate enzymes possessed lipase activity, as evidenced by their ability to hydrolyze both tributyrin (a short-chain triglyceride) and tricaprylin (a medium-chain triglyceride). The hydrolysis of tricaprylin, an insoluble medium-chain triacylglycerol, is particularly significant because it distinguishes true lipase activity from general esterase activity, which typically favors soluble, short-chain esters (Camacho-Ruiz et al., 2015; Mateos-Díaz et al., 2012). While the hydrolytic activity of these novel lipases was lower compared to the commercial CalB, this could be attributed to the partial purification of our candidate enzymes *versus* the high purity of commercial CalB. Further purification steps are expected to enhance observed activity. Additionally, the potential for enhancing their catalytic efficiency through protein engineering remains significant, opening avenues for future optimization.

The characterization of substrate specificity using pNP-esters revealed a strong preference for pNP-acetate (C2) across all three candidate enzymes. Further investigation with a wider range of substrates, including natural triacylglycerols, would provide a more complete understanding of their substrate specificity. The optimal temperatures for MGL-1 (50 °C), MGL-2 (50 °C), and MGL-3 (55 °C) are notably higher than those of most fungal and bacterial lipases, which typically range between 40 °C and 45 °C (Zhang et al., 2025). Such performance at elevated temperatures might make them suitable for industrial applications. The optimal pH values varied among the candidates (pH 7-9). MGL-3 showed optimal activity at pH 9 and complete inactivation at pH ≤5, indicating an alkaline preference.

The lipases’ ability to retain activity at elevated temperatures while functioning in alkaline conditions suggests their potential suitability as detergent additives (Gurkok & Ozdal, 2021). This is important because harsh conditions such as high pH and temperature, are common in this application. The observed variation in MGL-2 activity with different buffers at the same pH highlights the importance of buffer composition. Specific ions and ionic strength influence enzyme activity (Stoffel et al., 2025; Zhao et al., 2025). This underscores the necessity for optimizing buffer systems for specific applications to maximize enzymatic efficiency. Overall, these novel lipases represent promising candidates for industrial applications, particularly given their unique biochemical properties.

For applications involving industrial oils and fats such as biodiesel production, detergent formulations, and food processing, it is critical to evaluate lipase activity using actual triglyceride substrates. In this study, we assessed enzymatic activity using short-chain tributyrin and medium-chain tricaprylin. These offer only a partial view of true lipolytic potential. To better understand the enzymes’ applicability in industrial settings, future studies should incorporate long-chain and commercially relevant triglycerides. Examples include olive oil, coconut oil, or soybean oil. These more accurately reflect operational substrates (Tan et al., 2023; Vardar-Yel, Tütüncü & Sürmeli, 2024). Additionally, para-nitrophenyl (pNP) esters are widely used in enzymatic assays. This is due to their simplicity and ability to cover a broad range of chain lengths (C2–C16). However, their results might not accurately reflect performance in complex lipid systems. pNP esters are structurally simpler and significantly smaller than the long-chain triglycerides found in industrial oils. Nevertheless, lipases remain valuable biocatalysts in the synthesis of active pharmaceutical ingredients (Khanra, Ravichandiran & Swain, 2025; Simić et al., 2022). In this area, substrates are typically small, water-soluble molecules. This dual relevance underscores the importance of tailoring substrate selection to the intended application when characterizing lipase activity.

## Conclusions

This study successfully combined structural and sequence-based similarity searches, utilizing metagenomic resources, to identify novel lipases with low similarity to existing reference protein sequences. The integrated method reliably and effectively identified 711 putative novel lipases. This provides a robust starting point for further thorough investigation. This research expands the repertoire of lipases, which is particularly valuable for discovering novel catalysts with distinct properties.

Experimental validation confirmed the true lipase activity of three selected candidates (MGL-1, MGL-2, and MGL-3). These novel lipases exhibited thermostability (optimal temperatures of 50−55 °C) and alkaline activity (optimal pH 7-9). These characteristics underscore their potential for industrial applications, particularly in detergent formulations.

##  Supplemental Information

10.7717/peerj.20462/supp-1 Supplemental Information 1 Protein profile and partial purification summary of candidate lipases (A) Raw SDS-PAGE confirming the crude expression of lipase candidates. Lane M: protein marker (kDa); Lane 1: MGL-1; Lane 2: MGL-2; Lane 3: MGL-3; Lane 4: LipX. (B) SDS-PAGE profile of partially purified lipases. Lane M: protein molecular marker (kDa), Lane 1: MGL- 1, Lane 2: MGL-2, Lane 3: MGL-3. To enhance solubility and facilitate purification, the expressed target genes were engineered as fusion proteins. These constructs included an N-terminal maltose-binding protein (MBP) tag (42.5 kDa), the target lipase protein (estimated at 27.1 kDa for MGL-1, 28.7 kDa for MGL-2, and 27.4 kDa for MGL-3), and a C-terminal His tag (0.8 kDa). The expected molecular weight of the full fusion proteins is approximately 71 kDa. An arrow indicates the estimated molecular weight of the target lipase protein band. (C) Purification summary. The table summarizes the total protein content, total enzyme activity, specific activity, and purification fold for crude and partially purified samples of MGL-1, MGL-2, and MGL-3. The p-nitrophenyl hexanoate (*p* NP-C6) was used as the substrate for all enzyme activity measurements.

10.7717/peerj.20462/supp-2 Supplemental Information 2 Raw data for sequences and structural similarity information as generated by Foldseek

10.7717/peerj.20462/supp-3 Supplemental Information 3 Raw data of sequence similarity against the reference protein database

10.7717/peerj.20462/supp-4 Supplemental Information 4 Raw data: Lipase activity and substrate specificity analysis

10.7717/peerj.20462/supp-5 Supplemental Information 5 Raw data: Optimum pH and optimum temperature analysis

## Additional Information and Declarations

### Data Availability

The following information was supplied regarding data availability: The raw data are available in Supplemental Files.
