# Supplementary material for: Identification of novel metagenomic lipases through integrated structural and sequence-based analysis
Source: PeerJ. 2026 Feb 2;14:e20462. doi: 10.7717/peerj.20462 (PMC12875215; doi:10.7717/peerj.20462)
Supplement: Supplemental Information 1 — (A) Raw SDS-PAGE confirming the crude expression of lipase candidates. Lane M: protein marker (kDa); Lane 1: MGL-1; Lane 2: MGL-2; Lane 3: MGL-3; Lane 4: LipX. (B) SDS-PAGE profile of partially purified lipases. Lane M: protein molecular marker (kDa), Lane 1: MGL- 1, Lane 2: MGL-2, Lane 3: MGL-3. To enhance solubility and facilitate purification, the expressed target genes were engineered as fusion proteins. These constructs included an N-terminal maltose-binding protein (MBP) tag (42.5 kDa), the target lipase protein (estimated at 27.1 kDa for MGL-1, 28.7 kDa for MGL-2, and 27.4 kDa for MGL-3), and a C-terminal His tag (0.8 kDa). The expected molecular weight of the full fusion proteins is approximately 71 kDa. An arrow indicates the estimated molecular weight of the target lipase protein band. (C) Purification summary. The table summarizes the total protein content, total enzyme activity, specific activity, and purification fold for crude and partially purified samples of MGL-1, MGL-2, and MGL-3. The p-nitrophenyl hexanoate (p NP-C6) was used as the substrate for all enzyme activity measurements. [file peerj-14-20462-s001.pdf]

A

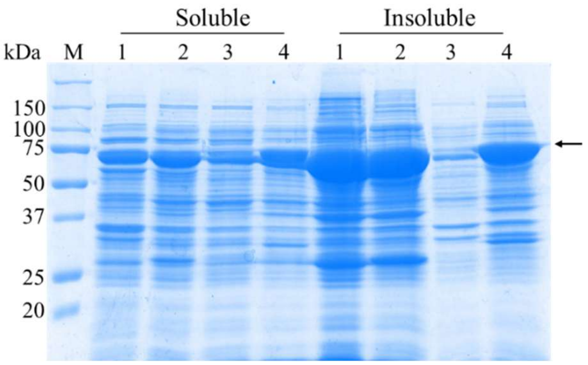

B

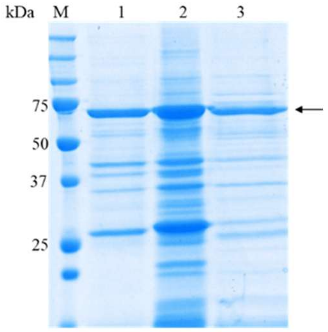

C

Lipase	Sample	Total protein (mg)	Enzyme activity (mU/mL)	Specific activity (mU/mg)	Purification (fold)
**MGL-1**	Crude	554.33 ± 17.69	32.20 ± 1.90	11.62 ± 0.69	1
	Partially purified	1.37 ± 0.08	15.21 ± 3.35	166.12 ± 36.62	14
**MGL-2**	Crude	374.46 ± 25.17	68.06 ± 2.69	36.35 ± 1.44	1
	Partially purified	5.16 ± 0.39	17.60 ± 1.36	51.13 ± 3.94	1.4
**MGL-3**	Crude	207.67 ± 17.23	9.65 ± 1.26	9.29 ± 1.21	1
	Partially purified	26.49 ± 0.74	52.54 ± 3.09	595.00 ± 35.03	64

Supplementary Fig.1 Protein profile and partial purification summary of candidate lipases. (A) Raw SDS-PAGE confirming the crude expression of lipase candidates. Lane M: protein marker (kDa); Lane 1: MGL-1; Lane 2: MGL-2; Lane 3: MGL-3; Lane 4: LipX. (B) SDS-PAGE profile of partially purified lipases. Lane M: protein molecular marker (kDa), Lane 1: MGL- 1, Lane 2: MGL-2, Lane 3: MGL-3. To enhance solubility and facilitate purification, the expressed target genes were engineered as fusion proteins. These constructs included an N-terminal maltose-binding protein (MBP) tag (42.5 kDa), the target lipase protein (estimated at 27.1 kDa for MGL-1, 28.7 kDa for MGL-2, and 27.4 kDa for MGL-3), and a C-terminal His tag (0.8 kDa). The expected molecular weight of the full fusion proteins is approximately 71 kDa. An arrow indicates the estimated molecular weight of the target lipase protein band. (C) Purification summary. The table summarizes the total protein content, total enzyme activity, specific activity, and purification fold for crude and partially purified samples of MGL-1, MGL-2, and MGL-3. The p-nitrophenyl hexanoate (*p*NP-C6) was used as the substrate for all enzyme activity measurements.
